# Functional and Structural Analysis of Predicted Proteins Obtained from *Homo sapiens'* Minisatellite 33.15-Tagged Transcript pAKT-45 Variants

**DOI:** 10.1155/2020/2562950

**Published:** 2020-05-23

**Authors:** Mohd Shahbaaz, Awad Saeed Al-Samghan, Arshi Malik, Sarah Afaq, Afaf S. Alwabli, Irfan Ahmad, Mostafa A. Hussien, Mohammad Zubair, Farha Fatima, Shamina Begum, Othman Alzahrani, Mohammed Tarique

**Affiliations:** ^1^South African Medical Research Council Bioinformatics Unit, South African National, Bioinformatics Institute, University of the Western Cape, South Africa; ^2^Laboratory of Computational Modeling of Drugs, South Ural State University, 76 Lenin Prospekt, Chelyabinsk, Russia 454080; ^3^Department of Family and Community Medicine, College of Medicine, King Khalid University, Abha 61421, Saudi Arabia; ^4^Department of Clinical Biochemistry, College of Medicine, King Khalid University, Abha, Saudi Arabia; ^5^Department of Biological Sciences, Faculty of Science, King Abdulaziz University, Jeddah 21589, Saudi Arabia; ^6^Department of Clinical Laboratory Sciences, College of Applied Medical Sciences, King Khalid University, Abha, Saudi Arabia; ^7^Research Center for Advanced Materials Science, King Khalid University, Abha, Saudi Arabia; ^8^Department of Chemistry, Faculty of Science, King Abdulaziz University, P.O. Box 80203 Jeddah 21589, Saudi Arabia; ^9^Department of Medical Microbiology, Faculty of Medicine, University of Tabuk, Tabuk 71491, Saudi Arabia; ^10^Department of Zoology, Faculty of Life Science, Aligarh Muslim University, Aligarh 202002, India; ^11^Department of Biology, Faculty of Science, University of Tabuk, Tabuk, Saudi Arabia; ^12^Genome and Biotechnology Unit, Faculty of Sciences, University of Tabuk, Tabuk, Saudi Arabia; ^13^Center for Interdisciplinary Research in Basic Sciences, Jamia Millia Islamia, Jamia Nagar, New Delhi 110025, India

## Abstract

The spermatozoa are transcriptionally dormant entities which have been recognized to be an archive of mRNA, coding for a variety of functionally crucial cellular proteins. This significant repository of mRNA is predicted to be associated with early embryogenesis and postfertilization. The mRNA transcripts which are tagged with minisatellites have been involved in the regulation of the gene functions as well as their organization. However, very little information is available regarding the expression of the transcripts tagged with minisatellites in spermatozoa. Therefore, in order to understand the functions and the conformational behavior of the proteins expressed from these minisatellite-tagged transcripts, we have performed a detailed *in silico* analysis using the sequences of the transcripts. The protein predicted from KF274549 showed the functionalities similar to uncharacterized C4orf26 proteins, while that obtained from KF274557 predicted to be a metallophosphoesterase. Furthermore, the structural folds in the structure of these predicted proteins were analyzed by using the homology modeling and their conformational behaviors in the explicit water conditions were analyzed by using the techniques of Molecular Dynamics (MD) simulations. This detailed analysis will facilitate the understanding of these proteins in the spermatozoon region and can be used for uncovering other attributes of the metabolic network.

## 1. Introduction

The ejaculated spermatozoa correspond to fixed terminally differentiated cells, which are devoid of the transcription as well as translation of the nuclear-encoded mRNAs. Consequently, the spermatozoa carry only the paternal genome to the ooplasm. The knowledge obtained from the discovery of a variety of soluble signaling molecules, transcription factors, and the molecules carried by the spermatozoa into the zygotic cytoplasm during fertilization has revolutionized this assessment (Saunders, Larman et al. [1–3]). Although the spermatozoa generally retain the transcriptionally dormant state, still the constituent mRNA transcripts present in its framework expressed into a variety of the transcription factors and these proteins may be involved in cell proliferation, signal transduction, regulation of sperm motility, acrosome reaction chromatin condensation, and capacitation [1, 2, 4–6]. Furthermore, the release of spermatozoal transcripts into the ooplasm is predicted to have a significant role during the process of fertilization and later stages. Generally, the spermatozoa contain around 3000–5000 mRNA transcripts, which may be involved in the expression and regulation of various constituent molecules [7–9].

The repeats present in the DNA sequences are dynamic elements of the genome and form the major portion of the satellites' regions and transposable components [10, 11]. These repeats are usually observed in the noncoding fragments of the genomes whereas a minor portion is preserved inside the transcriptome [12–14] and involved in gene regulation during gene silencing, transcription, and translation [9, 15, 16]. The mechanisms related to the expression and organization of these conserved repeats in the mammalian transcriptomes, predominantly in the spermatozoa, still remain undiscovered. Therefore, an *in silico* methodology was used for understanding the functions and conformational behavior of proteins expressed from such satellite DNA sections. The different functionalities were observed for the two proteins, KF274549 and KF274557, that were uncovered from *Homo sapiens'* minisatellite 33.15-tagged transcript sequence. The KF274549 showed similarities to the family of C4orf26 proteins, which are the group of hypothetical proteins. The hypothetical or uncharacterized proteins are the predicted polypeptides which do not have the experimental evidences at the biochemical levels [17–21]. The information regarding the functionalities of such proteins can be helpful in understanding the hidden mechanism behind the pathogenesis of a variety of microbial organisms [17–20]. Furthermore, the protein obtained from KF274557 showed the presence of metallophosphoesterase activities. These analyses can be useful in understanding the expression behavior of the satellite regions and how they are modulating the functionalities of other biomolecules present in the metabolic networks.

## 2. Material and Methods

The *in silico* methodology used for the analyses of the predicted proteins was obtained from KF274549 and KF274557. The primary stages involve the establishment of the phylogenetic relationships among the close homologs of the predicted proteins. Then, sequences of these proteins were utilized as the inputs in order to predict the conserved motifs and domains along with the functions they may perform in the metabolic network. We have also observed the availability of the possible interaction partners in the biological databases that may modulate their functionalities. Furthermore, the three dimensional (3-D) structures of these predicted proteins were modeled by using the X-ray crystal structures present in the publically available databases. We have also analyzed the conformational changes of these proteins in the explicit water environment by using the available methods of the MD simulations. The phases of the adopted methodology are explained here in details:

### 2.1. Protein Translation

The protein sequences for KF274549 and KF274557 were predicted by using the “Translate Tool” present in the Expasy server on the basis of standard genetic code and validated by using the “Translation” Module of the Discovery Studio 4.0 [22]. The Expasy's translate tools predicted six different protein sequences for each query DNA sequence. The BLASTx [23] was used for the selection of the most suitable translated protein sequence for further study. For KF274549, the output based on 3′-5′ Frame 2 was selected while for KF274557, the 5′-3′ Frame 3 was selected.

### 2.2. Sequence Analyses

A diverse range of bioinformatics was utilized for the functional analyses of the obtained proteins. In order to obtain the close homologs for the respective proteins, the sequence similarity tools such as BLASTp [23], HMMER [24], and HHpred [25] were utilized. For each sequence search, the proteins with low sequence identity (<20%) and query coverage (<50%) were excluded. Similarly, homologs with high sequence identities (>40%) were considered as the close homologs. Furthermore, the sequences of the close homologs were compared by using the multiple sequence alignment methods such as PRALINE [26]. The ClustalW method [27] was also utilized for inferring the information obtained from the alignment of the multiple sequences. On the basis of these alignments, the phylogenetic relationships were established by using the PHYLIP software package (http://evolution.genetics.washington.edu/phylip.html). The functional domains were annotated by using a variety of curated databases such as Pfam [28], SUPERFAMILY [29], PANTHER [30], SVMProt [31], CDART [32], SMART [33], InterPro [34], and ProtoNet [35]. Similarly, the conserved motifs in the sequences of KF274549 and KF274557 proteins were identified by using the MEME suite [36]. Moreover, the interaction partners in the metabolic network were predicted by using the STRING [37] database.

### 2.3. Structural Analyses

The understanding of the protein functionalities was further explored by analyzing their structural elements. The structures of the proteins were predicted by using the MODELLER [38] module of DS by satisfying the spatial restraints. The accuracy of the predicted models was evaluated using the Ramachandran plot. The topology of the generated models was analyzed by using the PDBsum [39], and the structural homologs were searched by using the Dali server [40]. Furthermore, the conformational behavior of the predicted protein was understood by performing the Molecular Dynamics (MD) simulations using the GROMACS 4.6.5 [41] software package. The proteins were solvated by using the SPC/E water model [42], and through the steepest descent algorithm, the energy minimization was performed with a convergence criterion of 0.005 kcal mol^−1^. The equilibration phase was carried out under NVT (constant volume) and NPT (constant pressure) ensemble conditions. The temperature of the system was maintained at 300 K by using the Berendsen weak coupling method in both ensemble conditions along with pressure which was maintained at 1 bar by utilizing the Parrinello-Rahman barostat in constant pressure ensemble. The final MD was produced by using the LINCS algorithm for the time scale of 200 ns. The knowledge extracted from the trajectory files was utilized for understanding the behavior of protein in the explicit solvent environment. The Root Mean Square Deviations (RMSD), radius of gyration (Rg), and Root Mean Square Fluctuations (RMSF) were analyzed.

## 3. Result and Discussion

The minisatellites are found to be involved in the regulation of gene expression, the unstable regions of the chromosomes, and the genome imprinting [7–9]. Yet, expression profiles and biological implication of their correlation with the coding regions still remain principally unresolved. In this study, we have performed an *in silico* analyses that enable us to understand the characteristic of translated protein obtained from the KF274549 and KF274557. The purpose of choosing these transcripts is explained in a previously published work [7–9]. The detailed analysis of each protein is discussed here separately.

### 3.1. KF274549 (T1)

**Figure 1:**
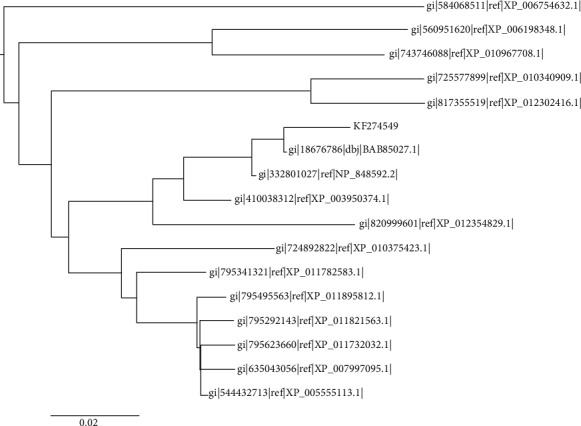
The phylogenetic analyses of T1 showing closeness to C4orf26 proteins.

**Figure 2:**
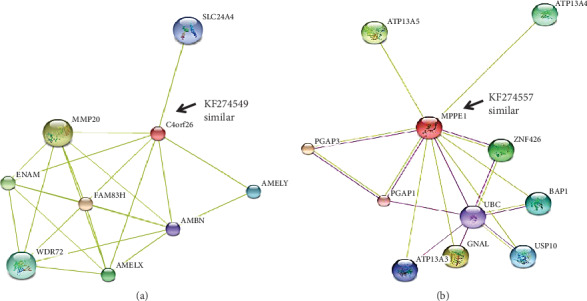
Predicted interaction partners of T1 and T2 in the metabolic network according to STRING database.

T1 showed high sequence similarities to uncharacterized C4orf26 proteins (Figure S1), with the highest similarity found with unnamed protein GI:18676786 (Figure 1), whereas the outcomes of Pfam and InterPro showed that the T1 belongs to the DUF4721, a family of protein with a domain of unknown functions. Similarly, PANTHER classifies the T1 into the family of uncharacterized proteins PTHR40376. The SMART server identifies that the T1 may belong to the superfamily of ubiquitin-like proteins. The proteins belonging to the ubiquitin-proteasome (ubiquitin-proteasome) system have an established role in the process of protein degradation in the human cell [43]. During spermatogenesis, the ubiquitination enzymes play a major role in the formation of the normal sperm by replacing histone with protamine [43]. Lately, the histone ubiquitin ligases were discovered to play crucial roles in several stages of spermatogenesis, such as DNA damage response, meiotic sex chromosome inactivation, and spermiogenesis [43]. Furthermore, the ProtoNet classifies the T1 into cluster 3965083, which is the collection of mammalian secreted proteins. The STRING database predicted that the T1 interact with protein member H, matrix metallopeptidase 20, enamelin, amelogenin, WD repeat domain 72, amelogenin, solute carrier family 24 (sodium/potassium/calcium exchanger), and ameloblastin (Figure 2(a)). In addition, we were able to observe three motifs in the sequence of KF274549 by using the MEME suite, namely, 47′-CNHRFPFQ, 119′-IKYPKHHLGRW, and 82′–SEGRET.

**Figure 3:**
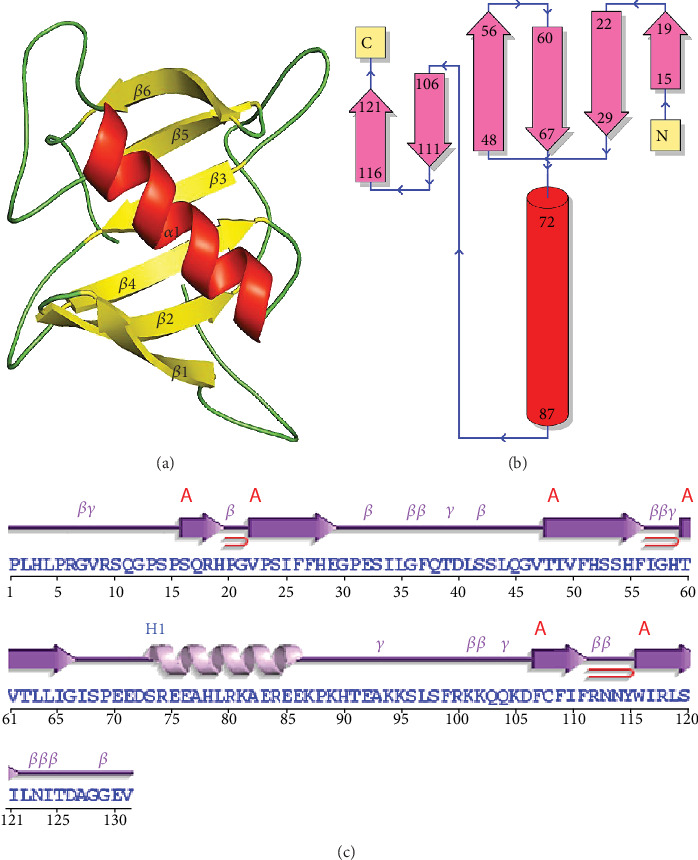
(a) The predicted model of T1 protein. (b, c) The topology generated by the PDBsum server on the basis of the modeled T1 protein.

**Figure 4:**
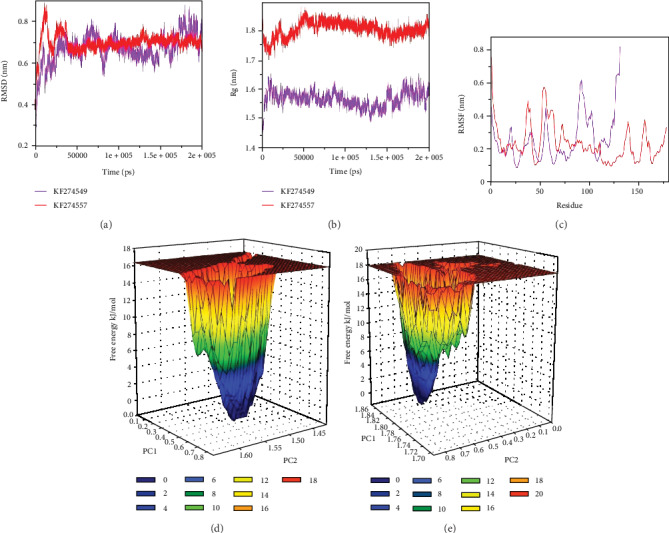
The outcomes obtained from the MD simulations for 200 ns time scale for both T1 and T2. (a) The plot showing the changes in the RMSD values of both the protein during the course of MD simulations. (b) The changes in the radius of gyrations (Rg) showing the variations observed in the compactness of the proteins. (c) The plot showing the fluctuations observed in the constituent residues. (d) The generated free energy landscape of T1. (e) The projected free energy landscape of T2 showing relative structure stability.

The structure of T1 was predicted by using the crystal structure of the hypothetical protein ORF126 (PDB ID-2X5R), which is involved in the molecular process of metal ion binding. The predicted structure contains one alpha and six beta strand secondary structural elements (Figure 3(a)). The stereochemical analyses on the basis of Ramachandran plots showed that 97.7% of the residues were present in the allowed region of the plot, which is indicative of the reliability of the predicted models. Similarly, PDBsum analyzed the presence of 1 sheet, 3 beta hairpins, 1 psi loop, 3 beta bulges, 6 strands, 1 helix, 16 beta turns, and 5 gamma turns in the topological description of the modeled structure (Figures 3(b) and 3(c)). The Dali server identified the structural homologs with functionalities as carbamoyl-phosphate synthetase large chain. Therefore, T1 may be involved in the pyrimidine biosynthesis in mammalian spermatozoa [44]. The topology of the predicted model was generated by using the OPLS all-atom force field. Then, it was solvated by using the SPCE water model and minimized for 1000 steps of steepest descent. The NVT and NPT equilibration each for 100 ps time scale was carried out. Finally, the protein was simulated for a 200 ns time scale. The trajectories of the simulations were analyzed by using GROMACS utilities. The RMSD values which provide the assessment regarding the system stability showed a continuous increase till 25 ns and become stable after 40 ns with fluctuations which were observed between 0.6 nm and 0.8 nm (Figure 4(a)). Similarly, the radius of gyration (Rg) is the measure of the compactness of the protein structures that were observed to be around 1.6 nm (Figure 4(b)). Furthermore, constituent residues showed relatively higher fluctuations around the residue count of 75-100 which correspond to the alpha-helix region of the predicted structure (Figure 4(c)). The free energy landscapes were projected and showed the presence of relatively unstable conformational behavior in T1 (Figure 4(d)).

### 3.2. KF274557 (T2)

**Figure 5:**
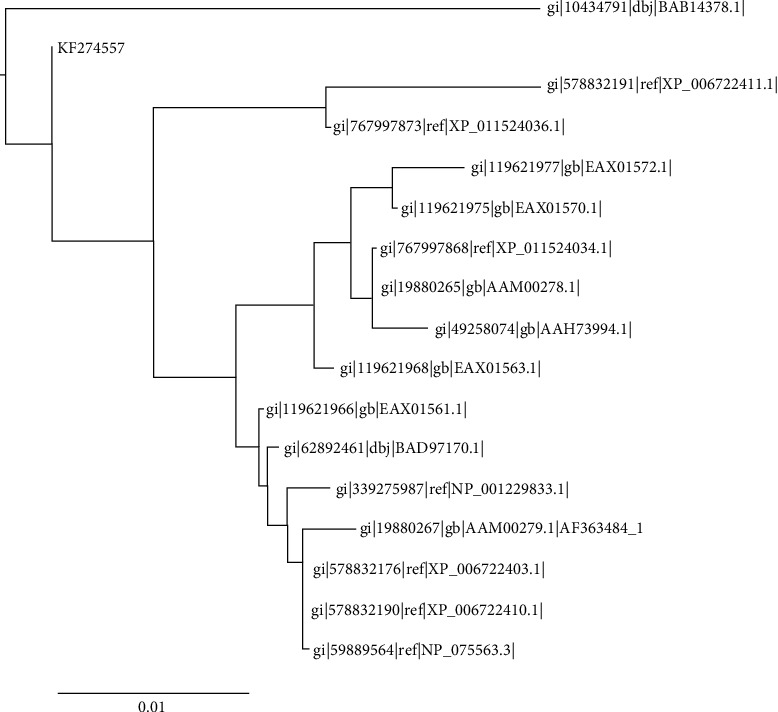
The outcomes of molecular phylogenetic analyses for T2 which showed higher homology towards metallophosphoesterases.

The sequence similarities for T2 showed high closeness to metallophosphoesterase (Figure S2) as well as showed similarities with a diversity of proteins (Figure 5). The outputs generated by CDART, SMART, and ProtoNet also validated the presence of metallophosphatase activities in T2. The mammalian phosphodiesterases in the spermatozoa were found to be significant for the capacitation and fertilization [45]. This category of the protein is also involved in the modulation of cyclic nucleotide cellular levels by catalyzing their degradation [46]. The cyclic nucleotides are involved in the regulation of sperm motility as well as acrosome reaction. The predicted interaction partners of T2 are post-GPI attachment to protein 3, guanine nucleotide-binding protein (G protein), zinc finger protein 426, ubiquitin-specific peptidase 10, ATPase-type 13A3, ubiquitin C, and post-GPI attachment to protein 1 (Figure 2(b)). Moreover, the MEME suite identified three motifs, namely, 18′-MNSDFGEQ, 119′-IYYLAIFQCNW, and 137′–SDGEQT.

**Figure 6:**
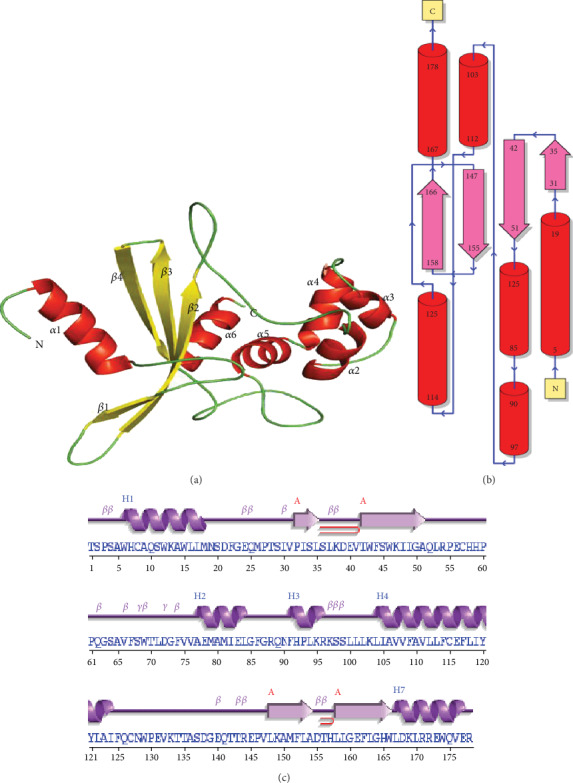
(a) The obtained 3-D structure of T2 protein by using homology modeling. (b, c) The PDBsum-based observed topology of the T2.

The three dimensional (3-D) structure of T2 was predicted by using the restriction endonuclease MspI (PDB ID-1SA3). The predicted structure of T2 contains the characteristic six alpha and four beta strands (Figure 6(a)). The modeled structure showed 98.3% of the constituent residues in the allowed region of the Ramachandran plot. In addition, the PDBsum identified 1 sheet, 1 beta alpha beta unit, 2 beta hairpins, 2 beta bulges, 4 strands, 7 helices, 5 helix-helix interacts, 19 beta turns, and 2 gamma turns in the predicted structure of T2 (Figures 6(b) and 6(c)). The structural homologs identified by the Dali server showed functionalities of type 2 restriction endonucleases and can be considered as significant for the sperm acrosome reaction [47]. Furthermore, the solvated model of T2 was minimized for 1200 steps of the steepest descent algorithm. After equilibration, the final MD simulations were executed for a 200 ns time scale. The dynamical analyses showed that RMSD values showed a continuous perturbation up to 40 ns, while after that, the values become constant at 0.7 nm (Figure 4(a)). Moreover, Rg values showed continuous fluctuations around 1.75–1.85 nm (Figure 4(b)), while constituent residues showed higher RMSF values in the residue region of 25-75 corresponding to the beta-sheet of the predicted structure (Figure 4(c)). Furthermore, the free energy landscapes showed a comparatively stable structural conformation of T2 (Figure 4(e)).

## 4. Conclusions

The current study identified the possible functions and conformational behavior associated with the proteins translated from *Homo sapiens*' minisatellite 33.15-tagged transcript pAKT-45 variants. The T1 was not showing any functional identities at the primary structure level as high similarities were observed with hypothetical proteins. Therefore, structure-based methods were utilized which predicted the T1 homology to carbamoyl-phosphate synthetase large chain, while the sequence and structure-based function of T2 was predicted with high confidence. Furthermore, the T2 showed a higher stability profile as compared to the T1 during the course of 200 ns MD simulations. This is attributed to the difference in the structural compactness of both the proteins. This study provides a better understanding of the behavior exhibited by the minisatellite regions at the protein level and their possible functionalities in the metabolic networks.

## Data Availability

The data used to support the findings of this study are included in the article.

## Supplementary Materials

Supplementary Materials Figure S1: the multiple sequence alignment showing high similarities of T1 with C4orf26 proteins. Figure S2: the multiple sequence alignment showing high similarities of T2 with metallophosphoesterase proteins.

## References

[1] Krawetz S. A. (2005). Paternal contribution: new insights and future challenges. *Nature Reviews Genetics*.

[2] Miller D., Ostermeier G. C., Krawetz S. A. (2005). The controversy, potential and roles of spermatozoal RNA. *Trends in Molecular Medicine*.

[3] Saunders C. M., Larman M. G., Parrington J. (2002). PLC zeta: a sperm-specific trigger of Ca(2+) oscillations in eggs and embryo development. *Development*.

[4] Lambard S., Galeraud-Denis I., Martin G., Levy R., Chocat A., Carreau S. (2004). Analysis and significance of mRNA in human ejaculated sperm from normozoospermic donors: relationship to sperm motility and capacitation. *Molecular Human Reproduction*.

[5] Miller D. (2000). Analysis and significance of messenger RNA in human ejaculated spermatozoa. *Molecular Reproduction and Development*.

[6] Wykes S. M., Visscher D. W., Krawetz S. A. (1997). Haploid transcripts persist in mature human spermatozoa. *Molecular Human Reproduction*.

[7] Srivastava J., Premi S., Pathak D. (2006). Transcriptional status of known and novel genes tagged with consensus of 33.15 repeat loci employing minisatellite-associated sequence amplification (MASA) and real-time PCR in water buffalo, Bubalus bubalis. *DNA and Cell Biology*.

[8] Toth G., Gaspari Z., Jurka J. (2000). Microsatellites in different eukaryotic genomes: survey and analysis. *Genome Research*.

[9] Vergnaud G., Denoeud F. (2000). Minisatellites: mutability and genome architecture. *Genome Research*.

[10] Charlesworth B., Sniegowski P., Stephan W. (1994). The evolutionary dynamics of repetitive DNA in eukaryotes. *Nature*.

[11] Jeffreys A. J., Royle N. J., Wilson V., Wong Z. (1988). Spontaneous mutation rates to new length alleles at tandem-repetitive hypervariable loci in human DNA. *Nature*.

[12] Bennett P. (2000). Demystified ... microsatellites. *Molecular Pathology*.

[13] Borstnik B., Pumpernik D. (2002). Tandem repeats in protein coding regions of primate genes. *Genome Research*.

[14] Jasinska A., Krzyzosiak W. J. (2004). Repetitive sequences that shape the human transcriptome. *FEBS Letters*.

[15] Li Y. C., Korol A. B., Fahima T., Nevo E. (2004). Microsatellites within genes: structure, function, and evolution. *Molecular Biology and Evolution*.

[16] Rocha E. P., Matic I., Taddei F. (2002). Over-representation of repeats in stress response genes: a strategy to increase versatility under stressful conditions?. *Nucleic Acids Research*.

[17] Shahbaaz M., Ahmad F., Hassan M. I. (2015). Structure-based function analysis of putative conserved proteins with isomerase activity from Haemophilus influenzae. *3 Biotech*.

[18] Shahbaaz M., Bisetty K., Ahmad F., Hassan M. I. (2015). Current advances in the identification and characterization of putative drug and vaccine targets in the bacterial genomes. *Current Topics in Medicinal Chemistry*.

[19] Shahbaaz M., Bisetty K., Ahmad F., Hassan M. I. (2015). Functional insight into putative conserved proteins of Rickettsia rickettsii and their virulence characterization. *Current Proteomics*.

[20] Shahbaaz M., Bisetty K., Ahmad F., Hassan M. I. (2015). Towards new drug targets? Function prediction of putative proteins of Neisseria meningitidis MC58 and their virulence characterization. *OMICS: A Journal of Integrative Biology*.

[21] Shahbaaz M., Hassan M. I., Ahmad F. (2013). Functional annotation of conserved hypothetical proteins from Haemophilus influenzae Rd KW20. *PLoS One*.

[22] Biovia D. S. (2013). *Discovery Studio Modeling Environment*.

[23] Altschul S. F., Gish W., Miller W., Myers E. W., Lipman D. J. (1990). Basic local alignment search tool. *Journal of Molecular Biology*.

[24] Finn R. D., Clements J., Eddy S. R. (2011). HMMER web server: interactive sequence similarity searching. *Nucleic Acids Research*.

[25] Soding J., Biegert A., Lupas A. N. (2005). The HHpred interactive server for protein homology detection and structure prediction. *Nucleic Acids Research*.

[26] Simossis V. A., Heringa J. (2005). PRALINE: a multiple sequence alignment toolbox that integrates homology-extended and secondary structure information. *Nucleic Acids Research*.

[27] Thompson J. D., Gibson T. J., Higgins D. G. (2002). Multiple sequence alignment using ClustalW and ClustalX. *Current Protocols in Bioinformatics*.

[28] Bateman A., Birney E., Cerruti L. (2002). The Pfam protein families database. *Nucleic Acids Research*.

[29] Gough J., Karplus K., Hughey R., Chothia C. (2001). Assignment of homology to genome sequences using a library of hidden Markov models that represent all proteins of known structure. *Journal of Molecular Biology*.

[30] Mi H., Muruganujan A., Casagrande J. T., Thomas P. D. (2013). Large-scale gene function analysis with the PANTHER classification system. *Nature Protocols*.

[31] Cai C. Z., Han L. Y., Ji Z. L., Chen X., Chen Y. Z. (2003). SVM-Prot: web-based support vector machine software for functional classification of a protein from its primary sequence. *Nucleic Acids Research*.

[32] Geer L. Y., Domrachev M., Lipman D. J., Bryant S. H. (2002). CDART: protein homology by domain architecture. *Genome Research*.

[33] Letunic I., Doerks T., Bork P. (2012). SMART 7: recent updates to the protein domain annotation resource. *Nucleic Acids Research*.

[34] Hunter S., Apweiler R., Attwood T. K. (2009). InterPro: the integrative protein signature database. *Nucleic Acids Research*.

[35] Rappoport N., Karsenty S., Stern A., Linial N., Linial M. (2012). ProtoNet 6.0: organizing 10 million protein sequences in a compact hierarchical family tree. *Nucleic Acids Research*.

[36] Bailey T. L., Boden M., Buske F. A. (2009). MEME SUITE: tools for motif discovery and searching. *Nucleic Acids Research*.

[37] Szklarczyk D., Franceschini A., Kuhn M. (2010). The STRING database in 2011: functional interaction networks of proteins, globally integrated and scored. *Nucleic Acids Research*.

[38] Eswar N., Webb B., Marti-Renom M. A. (2006). Comparative protein structure modeling using Modeller. *Current Protocols in Bioinformatics*.

[39] Laskowski R. A., Hutchinson E. G., Michie A. D., Wallace A. C., Jones M. L., Thornton J. M. (1997). PDBsum: a web-based database of summaries and analyses of all PDB structures. *Trends in Biochemical Sciences*.

[40] Holm L., Rosenstrom P. (2010). Dali server: conservation mapping in 3D. *Nucleic Acids Research*.

[41] Pronk S., Pall S., Schulz R. (2013). GROMACS 4.5: a high-throughput and highly parallel open source molecular simulation toolkit. *Bioinformatics*.

[42] Zielkiewicz J. (2005). Structural properties of water: comparison of the SPC, SPCE, TIP4P, and TIP5P models of water. *The Journal of Chemical Physics*.

[43] Sheng K., Liang X., Huang S., Xu W. (2014). The role of histone ubiquitination during spermatogenesis. *BioMed Research International*.

[44] Carrey E. A., Dietz C., Glubb D. M., Loffler M., Lucocq J. M., Watson P. F. (2002). Detection and location of the enzymes of de novo pyrimidine biosynthesis in mammalian spermatozoa. *Reproduction*.

[45] Baxendale R. W., Fraser L. R. (2005). Mammalian sperm phosphodiesterases and their involvement in receptor-mediated cell signaling important for capacitation. *Molecular Reproduction and Development*.

[46] Bajpai M., Fiedler S. E., Huang Z. (2006). AKAP3 selectively binds PDE4A isoforms in bovine spermatozoa. *Biology of Reproduction*.

[47] Meizel S. (1984). The importance of hydrolytic enzymes to an Exocytotic event, the mammalian sperm acrosome reaction. *Biological Reviews*.

