# Supplementary material for: Functional and Structural Analysis of Predicted Proteins Obtained from Homo sapiens' Minisatellite 33.15-Tagged Transcript pAKT-45 Variants
Source: Biomed Res Int. 2020 May 23;2020:2562950. doi: 10.1155/2020/2562950 (PMC7273396; doi:10.1155/2020/2562950)
Supplement: Supplementary Materials — Figure S1: the multiple sequence alignment showing high similarities of T1 with C4orf26 proteins. Figure S2: the multiple sequence alignment showing high similarities of T2 with metallophosphoesterase proteins. [file 2562950.f1.pdf]

Unconserved 0 1 2 3 4 5 6 7 8 9 10 **Conserved**

```
                 .........10 .........20 .........30 .........40 .........50
KF274549         ---------- ---------- ---------- ---------- ---------TS
gi_18676786_dbj  MARRHCFSYW LLVCWLVVTV AEGQEEVFTL PGDSQNNADA TDCQIFTLTP
gi_332801027_re  MARRHCFSYW LLVCWLVVTV AEGQEEVFTP PGDSQNNADA TDCQIFTLTP
gi_410038312_re  MARRHCFSYW LLVCWLVVTV AEGQEEVFTP PGDSQNNADA TDCQIFTLTP
gi_820999601_re  MAHRHRFSYW LLVCWLVVTV AEGQEEVFTP PGDSQNNADA TDCQIFTLTP
gi_795341321_re  MARRHCFSYW LLVCWLVVTV AEGQEEVFTP PGDSQNNANP TDCRIFTLTP
gi_724892822_re  MARRHCFSYS LLVCWLVVTV AEGQEEIFTP PGDSQNNANP TDCQIFTLTP
gi_795495563_re  MARRHCFSYW LLVCWLAVTV AEGQEEVFTP PGDSQNNANP TDCQIFTLTP
gi_544432713_re  MARRHCFSYW LLVCWLAVTV AEGQEEVFTP PGDSQNNANP TDCQIFTLTP
gi_795292143_re  MARRHCFSYW LLVCWLAVTV AEGQEEVFTP PGDSQNNANP TDCQIFTLTP
gi_635043056_re  MARRHCFSYW LLVCWLAVTV AEGQEEVFTP PGDSQNNANP TDCQIFTLTP
gi_795623660_re  MARRHCFSYW LLVCWLAVTV AEGQEEVFTP PGDSQNNANP TDCQIFTLTP
gi_725577899_re  MARRHCFSYW LLVCWLVVTV AEGQEEVFTP RGDSENNADH TDCQVFTLTP
gi_817355519_re  MARRHCFSYW LLVCWLVVTV AEGQEEVFTP PGDSENNADH TDCQIFTLTP
gi_560951620_re  MAHRLSYSYC LLVCWLVVTV AEGQEGVVTP PGDSQHNADP TDCQIFTLTP
gi_743746088_re  MAHRLSYSYC LLVCWLVVTV AEGQEGVVTP PGDSQHNADP TDCQIFTLTP
gi_584068511_re  MAHRLCLSCW LLVCWLVVMV AEGQ--VVTP PGGSQTEG-P TDCQIFTLTP
Consistency      8868667876 8888886888 8888768687 7888878855 88888888*9

                 .........60 .........70 .........80 .........90 ........100
KF274549         PPAPRSPVTR AQPITKTPRC PFHFFP-R-- --RPRIHFRF PNRPFVPSRC
gi_18676786_dbj  PPAPRSPVTR AQPITKTPRC PFHFFP-R-- --RPRIHFRF PNRPFVPSRC
gi_332801027_re  PPAPRSPVTR AQPITKTPRC PFHFFP-R-- --RPRIHFRF PNRPFVPSRC
gi_410038312_re  PPAPRSPVTR AQPITKTPRC PFHFFP-R-- --RPRIHFRF PNRPFLPSRC
gi_820999601_re  PPARRSPVTT FQPITKTPRC PFHFFP-R-- --RPRIHFRF PNRPFLPSRC
gi_795341321_re  PPATRSPVTR VQPITKTPRC PFHFFP-R-- --RPRIHFRF PNRPFLPSRC
gi_724892822_re  PPTTRSPVTR VQPITKTPRC PFHFFP-R-- --RPRIHFRF PNRPFLPSRC
gi_795495563_re  PPATRSPVTR GQPITKTPRC PFHFFP-R-- --RPRIYFRF PNRPFLPSRC
gi_544432713_re  PPATRSPVTR VQPITKTPRC PFHFFP-R-- --RPRIYFRF PNRPFLPSRC
gi_795292143_re  PPATRSPVTR VQPITKTPKC PFHFFP-R-- --RPRIYFRF PNRPFLPSRC
gi_635043056_re  PPATRSPVTR VQPITKTPRC PFHFFP-R-- --RPRIYFRF PNRPFLPSRC
gi_795623660_re  PPATRSPVTR VQPITKTPRC PFHFFP-R-- --RPRIYFRF PNRSFLPSRC
gi_725577899_re  PPVTRSPVTR AQPVTKTPRC PFLFFP-RRF PQRPRVHFRF PNRPFLPSRC
gi_817355519_re  PPITRSPVTR AQPITKPPRC PFLFFP-RRF PQRPRVHFQF PNRPFLPSRC
gi_560951620_re  PPTTRKPVTR IQPITRTPRC PLHFFPPR-- --CPRVHLRF PYRPFHPPTC
gi_743746088_re  PPTTRKPVTR IQPITRTPRC PLHFFPPR-- --CPRVHLRF PYRPFHPPTC
gi_584068511_re  PPATRRPVTR IQPITRTPKC PFHFFPTR-- --RPRVQIRF PNRPFLPPRC
Consistency      **76*8***9 6**9*89*9* *88***0*00 007**9789* *8*9*7*78*

                 ........110.........120.........130.........140.........150
KF274549         NHRFPFQPFY WPHRYLT--Y RYFPRRRLQR GSSSEESEGR ET--QTYSKK
gi_18676786_dbj  NHRFPFQPFY WPHRYLT--Y RYFPRRRLQR GSSSEES--- ----------
gi_332801027_re  NHRFPFQPFY WPHRYLT--Y RYFPRRRLQR GSSSEES--- ----------
gi_410038312_re  NHRFPFQPFY WPHRHLT--Y GYFPRRRLQR GSSSEES--- ----------
gi_820999601_re  NHRFPFPSFY WPHRHLT--Y RYFPRRRLQR GSSSEES--- ----------
gi_795341321_re  NHRFPFQPFY WPHRRLTP-Y RYLPRRRLQR GSSSEES--- ----------
gi_724892822_re  NHRFPLQRFY WPHRRLTP-Y RYFPRRRLQR GSSSEES--- ----------
gi_795495563_re  NHRFPFQPFY WPHRRLTP-Y RYFPRRRLQR GSSSEES--- ----------
gi_544432713_re  NHRFPFQPFY WPHRRLTP-Y RYFPRRRLQR GSSSEES--- ----------
gi_795292143_re  NHRFPFQPFY WPHRRLTP-Y RYFPRRRLQR GSSSEES--- ----------
gi_635043056_re  NHHFPFQPFY WPHRRLTP-Y RYFPRRRLQR GSSSEES--- ----------
gi_795623660_re  NHRFPFQPFY WPHRRLTP-Y RYFPRRRLQR GSSSEES--- ----------
gi_725577899_re  NYRFPFQPFY WPHRRLTP-G RYFLRRRLQR GSSSEEI--- ----------
gi_817355519_re  NYRFPFQPFY WPHRRLNP-G -RYFLRRLQR GSSSEEI--- ----------
gi_560951620_re  NHRFQIHPFF WPHRRLPPHY RYFPRRPLWR GSSSEESRAK REAPDILKEK
gi_743746088_re  NHRFQIHPFF WPHRRLPPHY RYFPRRPLWR GSSSEESRAK REAPNILKEK
gi_584068511_re  NHRFHYRPFF RPNNRLSQHY NYFPRRRLWR GSSSEERRGK REAPNLLNQR
Consistency      *89*7778*8 8*996*7408 79879*8*7* ******7000 0000000000

                 ........160.........170.........180.........190.........200
KF274549         KPILQ-KKAT KRF--LFYL- --SKLKL--- --LDLKIKYP KHH-L-GRWR
gi_18676786_dbj  ---------- ---------- ---------- ---------- ----------
gi_332801027_re  ---------- ---------- ---------- ---------- ----------
gi_410038312_re  ---------- ---------- ---------- ---------- ----------
gi_820999601_re  ---------- ---------- ---------- ---------- ----------
gi_795341321_re  ---------- ---------- ---------- ---------- ----------
gi_724892822_re  ---------- ---------- ---------- ---------- ----------
gi_795495563_re  ---------- ---------- ---------- ---------- ----------
gi_544432713_re  ---------- ---------- ---------- ---------- ----------
gi_795292143_re  ---------- ---------- ---------- ---------- ----------
gi_635043056_re  ---------- ---------- ---------- ---------- ----------
```

```
gi_795623660_re ---------- ---------- ---------- ---------- ----------
gi_725577899_re ---------- ---------- ---------- ---------- ----------
gi_817355519_re ---------- ---------- ---------- ---------- ----------
gi_560951620_re KPMMGRWGII RKGPTLGKLE DGSVLYLNLP PSLDLSSRRL AHLPLFTSCS
gi_743746088_re KPMM------ RR-------- ----VKL--- -----SGRAH AQHA------
gi_584068511_re RSIP-----P KRL------- ---------- ---------- ----------
Consistency     0000000000 0000000000 0000000000 0000000000 0000000000

                ..
KF274549        G-
gi_18676786_dbj --
gi_332801027_re --
gi_410038312_re --
gi_820999601_re --
gi_795341321_re --
gi_724892822_re --
gi_795495563_re --
gi_544432713_re --
gi_795292143_re --
gi_635043056_re --
gi_795623660_re --
gi_725577899_re --
gi_817355519_re --
gi_560951620_re AL
gi_743746088_re --
gi_584068511_re --
Consistency     00
```

Figure S1

Unconserved 0 1 2 3 4 5 6 7 8 9 10 Conserved

```
KF274557           ---------- ---------- ---------- --TSPSAWHC AQSWKAWLLM
gi_767997873_re    WPEVKTTASD GEQTTREPVL KAMFLADTHL LGEFLGHWLD KLR-REWQME
gi_767997868_re    WPEVKTTASD GEQTTREPVL KAMFLADTHL LGEFLGHWLD KLR-REWQME
gi_19880265_gb_    WPEVKTTASD GEQTTREPVL KAMFLADTHL LGEFLGHWLD KLR-REWQME
gi_119621975_gb    WPEVKTTASD GEQTTREPVL KAMFLADTHL LGEFLGHWLD KLR-REWQME
gi_119621968_gb    WPEVKTTASD GEQTTREPVL KAMFLADTHL LGEFLGHWLD KLR-REWQME
gi_49258074_gb_    WPEVKTTASD GEQTTREPVL KAMFLADTHL LGEFLGHWLD KLR-REWQME
gi_339275987_re    WPEVKTTASD GEQTTREPVL KAMFLADTHL LGEFLGHWLD KLR-REWQME
gi_119621966_gb    WPEVKTTASD GEQTTREPVL KAMFLADTHL LGEFLGHWLD KLR-REWQME
gi_578832176_re    WPEVKTTASD GEQTTREPVL KAMFLADTHL LGEFLGHWLD KLR-REWQME
gi_59889564_ref    WPEVKTTASD GEQTTREPVL KAMFLADTHL LGEFLGHWLD KLR-REWQME
gi_62898461_dbj    WPEVKTTASD GEQTTREPVL KAMFLADTHL LGEFLGHWLD KLR-REWQME
gi_19880267_gb_    WPEVKTTASD GEQTTREPVL KAMFLADTHL LGEFLGHWLD KLR-REWQME
gi_578832190_re    WPEVKTTASD GEQTTREPVL KAMFLADTHL LGEFLGHWLD KLR-REWQME
gi_119621977_gb    WPEVKTTASD GEQTTREPVL KAMFLADTHL LGEFLGHWLD KLR-REWQME
gi_578832192_re    WPEVKTTASD GEQTTREPVL KAMFLADTHL LGEFLGHWLD KLR-REWQME
gi_10434791_dbj    WPEVKTTASD GEQTTREPVL KAMFLADTHL LGEFLGHWLD KLR-REWQME
Consistency        8888888888 8888888888 8888888888 889989*88 999099*999

                   .........110.........120.........130.........140.........150
KF274557           NSDFGEQMPT SLVP-ISLSL KDEV------ TWFSWKIIGA QLRPECHHPP
gi_767997873_re    RA-FQTAL-W LLQPEVVFIL GDIFDEGKWS TPEAWADDVE RFQ-KMFRHP
gi_767997868_re    RA-FQTAL-W LLQPEVVFIL GDIFDEGKWS TPEAWADDVE RFQ-KMFRHP
gi_19880265_gb_    RA-FQTAL-W LLQPEVVFIL GDIFDEGKWS TPEAWADDVE RFQ-KMFRHP
gi_119621975_gb    RA-FQTAL-W LLQPEVVFIL GDIFDEGKWS TPEAWADDVE RFQ-KMFRHP
gi_119621968_gb    RA-FQTAL-W LLQPEVVFIL GDIFDEGKWS TPEAWADDVE RFQ-KMFRHP
gi_49258074_gb_    RA-FQTAL-W LLQPEVVFIL GDIFDEGKWS TPEAWADDVE RFQ-KMFRHP
gi_339275987_re    RA-FQTAL-W LLQPEVVFIL GDIFDEGKWS TPEAWADDVE RFQ-KMFRHP
gi_119621966_gb    RA-FQTAL-W LLQPEVVFIL GDIFDEGKWS TPEAWADDVE RFQ-KMFRHP
gi_578832176_re    RA-FQTAL-W LLQPEVVFIL GDIFDEGKWS TPEAWADDVE RFQ-KMFRHP
gi_59889564_ref    RA-FQTAL-W LLQPEVVFIL GDIFDEGKWS TPEAWADDVE RFQ-KMFRHP
gi_62898461_dbj    RA-FQTAL-W LLQPEVVFIL GDIFDEGKWS TPEAWADDVE RFQ-KMFRHP
gi_19880267_gb_    RA-FQTAL-W LLQPEVVFIL GDIFDEGKWS TPEAWADDVE RFQ-KMFRHP
gi_578832190_re    RA-FQTAL-W LLQPEVVFIL GDIFDEGKWS TPEAWADDVE RFQ-KMFRHP
gi_119621977_gb    RA-FQTAL-W LLQPEVVFIL GDIFDEGKWS TPEAWADDVE RFQ-KMFRHP
gi_578832192_re    RA-FQTAL-W LLQPEVVFIL GDIFDEGKWS TPEAWADDVE RFQ-KMFRHP
gi_10434791_dbj    RA-FQTAL-W LLQPEVVFIL GDIFDEGKWS TPEAWADDVE RFQ-KMFRHP
Consistency        990*99990 9 9*9*89999* 9*89888888 *889*98889 9990999999*

                   .........160.........170.........180.........190.........200
KF274557           QGSAVFSWT- -LD-GF---- VVAEMAMIEL GFGRQNFHPL KRKSSLLLKL
gi_767997873_re    SHVQLKVVAG NHDIGFHYEM NTYKVERFEK VFSSERLFSW KGINFVMVNS
gi_767997868_re    SHVQLKVVAG NHDIGFHYEM NTYKVERFEK VFSSERLFSW KGINFVMVNS
gi_19880265_gb_    SHVQLKVVAG NHDIGFHYEM NTYKVERFEK VFSSERLFSW KGINFVMVNS
gi_119621975_gb    SHVQLKVVAG NHDIGFHYEM NTYKVERFEK VFSSERLFSW KGINFVMVNS
gi_119621968_gb    SHVQLKVVAG NHDIGFHYEM NTYKVERFEK VFSSERLFSW KGINFVMVNS
gi_49258074_gb_    SHVQLKVVAG NHDIGFHYEM NTYKVERFEK VFSSERLFSW KGINFVMVNS
gi_339275987_re    SHVQLKVVAG NHDIGFHYEM NTYKVERFEK VFSSERLFSW KGINFVMVNS
gi_119621966_gb    SHVQLKVVAG NHDIGFHYEM NTYKVERFEK VFSSERLFSW KGINFVMVNS
gi_578832176_re    SHVQLKVVAG NHDIGFHYEM NTYKVERFEK VFSSERLFSW KGINFVMVNS
gi_59889564_ref    SHVQLKVVAG NHDIGFHYEM NTYKVERFEK VFSSERLFSW KGINFVMVNS
gi_62898461_dbj    SHVQLKVVAG NHDIGFHYEM NTYKVERFEK VFSSERLFSW KGINFVMVNS
gi_19880267_gb_    SHVQLKVVAG NHDIGFHYEM NTYKVERFEK VFSSERLFSW KGINFVMVNS
gi_578832190_re    SHVQLKVVAG NHDIGFHYEM NTYKVERFEK VFSSERLFSW KGINFVMVNS
gi_119621977_gb    SHVQLKVVAG NHDIGFH--- ---------- ---------- --YDFVMVNS
gi_578832192_re    SHVQLKVVAG NHDIGFHYEM NTYKVERFEK VFSSERLFSW KGINFVMVNS
gi_10434791_dbj    SHVQLKVVAG NHDIGFHYEM NTYKVERFEK VFSSERLFSW KGINFVMVNS
Consistency        9999989898 88*8**8777 7878888887 7888888887 8788999999

                   .........210.........220.........230.........240.........250
KF274557           IAV------- ----VFAVLL F--------- ---------- ----------
gi_767997873_re    VALNGDGC-G ICSETEAELI EVSHRLNCSR EG-------- ---------S
gi_767997868_re    VALNGDGC-G ICSETEAELI EVSHRLNCSR EQARGSSRCG PGPLLPTSAP
gi_19880265_gb_    VALNGDGC-G ICSETEAELI EVSHRLNCSR E-ARGSSRCG PGPLLPTSAP
gi_119621975_gb    VALNGDGC-G ICSETEAELI EVSHRLNCSR E-ARGSSRCG PGPLLPTSAP
gi_119621968_gb    VALNGDGC-G ICSETEAELI EVSHRLNCSR E-ARGSSRCG PGPLLPTSAP
gi_49258074_gb_    VALNGDGC-G ICSETEAELI EVSHRLNCSR EQARGSSRCG PGPLLPTSAP
gi_339275987_re    VALNGDGC-G ICSETEAELI EVSHRL---- ---------- ----------
gi_119621966_gb    VALNGDGC-G ICSETEAELI EVSHRLNCSR EQARGSSRCG PGPLLPTSAP
gi_578832176_re    VALNGDGC-G ICSETEAELI EVSHRLNCSR EQARGSSRCG PGPLLPTSAP
gi_59889564_ref    VALNGDGC-G ICSETEAELI EVSHRLNCSR EA-RGSSRCG PGPLLPTSAP
gi_62898461_dbj    VALNGDGC-G ICSETEAELI EVSHRLNCSR EA-RGSSRCG PGPLLPTSAP
gi_19880267_gb_    VALNGDGC-G ICSETEAELI EVSHRLNCSR EA-RGSSRCG PGPLLPTSAP
gi_578832190_re    VALNGDGC-G ICSETEAELI EVSHRLN--- ---------- ----------
gi_119621977_gb    VALNGDGC-G ICSETEAELI EVSHRLNCSR EQARGSSRCG PGPLLPTSAP
gi_578832192_re    VALNGDGC-G ICSETEAELI EVSHRLNCSR EQARGSSRCG PGPLLPTSAP
gi_10434791_dbj    VALNGMAVAS ALKQKQSSLK FLTD------ ---------- ----------
```

```
Consistency      9*98878808 888888898*8 8888776555 5124444444 4444444445

                 .........260.........270.........280.........290.........300
KF274557         ---------- -----C---- ---------- ---------- ----EFLIYY
gi_767997873_re  ITPTD----- ---------- ---------- ---------- ----------
gi_767997868_re  VLLQHYPLYR RSDANCSGED AAPAEERDIP FKENYDVLSR EASQK-LLWW
gi_19880265_gb_  VLLQHYPLYR RSDANCSGED AAPAEERDIP FKENYDVLSR EASQK-LLWW
gi_119621975_gb  VLLQHYPLYR RSDANCSGED AAPPEERDIP FKENYDVLSR EASQK-LLWW
gi_119621968_gb  VLLQHYPLYR RSDANCSGED AAPPEERDIP FKENYDVLSR EASQK-LLWW
gi_49258074_gb_  VLLQHYPLYR RSDANCSGED AAPAEERDIP FKENYDVLSR EASQK-LLWW
gi_339275987_re  ---------- ---------- ---------- ---------- NCSRE-LLWW
gi_119621966_gb  VLLQHYPLYR RSDANCSGED AAPPEERDIP FKENYDVLSR EASQK-----
gi_578832176_re  VLLQHYPLYR RSDANCSGED AAPAEERDIP FKENYDVLSR EASQK-LLWW
gi_59889564_ref  VLLQHYPLYR RSDANCSGED AAPAEERDIP FKENYDVLSR EASQK-LLWW
gi_62898461_dbj  VLLQHYPLYR RSDANCSGED AAPPEERDIP FKENYDVLSR EASQK-LLWW
gi_19880267_gb_  VLLQHYPLYR RSDANCSGED AAPAEERDIP FKENYDVLSR EASQK-LLWW
gi_578832190_re  CSREHYPLYR RSDANCSGED AAPAEERDIP FKENYDVLSR EASQK-LLWW
gi_119621977_gb  VLLQHYPLYR RSDANCSGED AAPPEERDIP FKENYDVLSR EASQK-----
gi_578832192_re  VLLQHYPLYR RSDANCSGED AAPAEERDIP FKENYDVLSR EASQKERSDG
gi_10434791_dbj  ---------- ---------- ---------- ---------- ----------
Consistency      5555555555 5555565555 5553555555 5555555555 6666605444

                 .........310.........320.........330.........340.........350
KF274557         L---AIFQ-- ----CNW--- ---PEVKTTA SDGEQTTREP VLK-------
gi_767997873_re  ---------- ---------- ---------- ---------- ----------
gi_767997868_re  LQPRLVLSGH THSACEVHHG GRVPELSVPS FSWRNRNNPS FIMGTDA---
gi_19880265_gb_  LQPRLVLSGH THSACEVHHG GRVPELSVPS FSWRNRNNPS FIMGTDA---
gi_119621975_gb  LQPRLVLSGH THSACEVHHG GRVPELSVPS FSWRNRNNPS FIMGTDA---
gi_119621968_gb  LQPRLVLSGH THSACEVHHG GRVPELSVPS FSWRNRNNPS FIMVT-----
gi_49258074_gb_  LQPRLVLIGH THSACEVHHG GRVPELSVPS FSWRNRNNPS FIMGTDA---
gi_339275987_re  LQPRLVLSGH THSACEVHHG GRVPELSVPS FSWRNRNNPS FIMGSITPTD
gi_119621966_gb  --PRLVLSGH THSACEVHHG GRVPELSVPS FSWRNRNNPS FIMGSITPTD
gi_578832176_re  LQPRLVLSGH THSACEVHHG GRVPELSVPS FSWRNRNNPS FIMGSITPTD
gi_59889564_ref  LQPRLVLSGH THSACEVHHG GRVPELSVPS FSWRNRNNPS FIMGSITPTD
gi_62898461_dbj  LQPRLVLSGH THSACEVHHG GRVPELSVPS FSWRNRNNPS FIMGSITPTD
gi_19880267_gb_  LQPRLVLSGH THSACEVHHG GRVPELSVPS FSWRNRNNPS FIMGSITPTD
gi_578832190_re  LQPRLVLSGH THSACEVHHG GRVPELSVPS FSWRNRNNPS FIMGSITPTD
gi_119621977_gb  --PRLVLSGH THSACEVHHG GRVPELSVPS FSWRNRNNPS FIMGTDA---
gi_578832192_re  QRPSAER--- ---------- ---------- ---------- ----------
gi_10434791_dbj  ---------- ---------- ---------- ---------- ----------
Consistency      5466666555 5555665555 5556666666 5656666666 6664423111

                 .........360.........370.........380.........390.........400
KF274557         ---------- --AMFLADTH LLGEFLGHWL D-------KL RREWQVER--
gi_767997873_re  YTLSKCYLPR EDVVLIIYCG VVGFLVVLTL THFGLLASPF LSGLNLLGKR
gi_767997868_re  ---------- ---------- ---------- ---------- ----------
gi_19880265_gb_  ---------- ---------- ---------- ---------- ----------
gi_119621975_gb  ---------- ---------- ---------- ---------- ----------
gi_119621968_gb  ---------- ---------- ---------- ---------- ----------
gi_49258074_gb_  ---------- ---------- ---------- ---------- ----------
gi_339275987_re  YTLSKCYLPR EDVVLIIYCG VVGFLVVLTL THFGLLASPF LSGLNLLGKR
gi_119621966_gb  YTLSKCYLPR EDVVLIIYCG VVGFLVVLTL THFGLLASPF LSGLNLLGKR
gi_578832176_re  YTLSKCYLPR EDVVLIIYCG VVGFLVVLTL THFGLLASPF LSGLNLLGKR
gi_59889564_ref  YTLSKCYLPR EDVVLIIYCG VVGFLVVLTL THFGLLASPF LSGLNLLGKR
gi_62898461_dbj  YTLSKCYLPR EDVVLIIYCG VVGFLVVLTL THFGLLASPF LSGLNLLGKR
gi_19880267_gb_  YTLSKCYLPR EDVVLIIYCG EVGFLVVLTL THFGLLASPF LSGLNLLGKR
gi_578832190_re  YTLSKCYLPR EDVVLIIYCG VVGFLVVLTL THFGLLASPF LSGLNLLGKR
gi_119621977_gb  ---------- ---------- ---------- ---------- ----------
gi_578832192_re  ---------- ---------- ---------- ---------- ----------
gi_10434791_dbj  ---------- ---------- ---------- ---------- ----------
Consistency      2222222222 2222222222 1222222222 2222222222 2222222222
```

Figure S2
